# Effect of the application of an edible film with turmeric (C*urcuma longa* L.) on the oxidative stability of meat

**DOI:** 10.1002/fsn3.1728

**Published:** 2020-07-11

**Authors:** Hylenne Bojorges, M. A. Ríos‐Corripio, Aleida S. Hernández‐Cázares, Juan Valente Hidalgo‐Contreras, Adriana Contreras‐Oliva

**Affiliations:** ^1^ Colegio de Postgraduados ‐ Campus Córdoba. Km. 348 Carretera Federal Córdoba‐Veracruz Amatlán de los Reyes Veracruz México; ^2^ CONACYT–Colegio de Postgraduados ‐ Campus Córdoba. Km. 348 Carretera Federal Córdoba–Veracruz Amatlán de los Reyes Veracruz México

**Keywords:** beef, chicken meat, edible film, pork, turmeric

## Abstract

The aim of this study was to develop an edible alginate‐based film produced with turmeric (EFT), as an active compound, and evaluate its antioxidant capacity for application in fresh pork loin, beef loin, and chicken breast. The EFT was characterized by barrier parameters, color, and mechanical, structural, and antioxidant properties. Meat samples with and without EFT were stored at 4°C and analyzed at 2‐day intervals. The meat samples with EFT showed significant differences (*p* < .05) in color (CIE L*a*b*) and exhibited lower TBARS values compared with those without EFT. The addition of turmeric in the film, besides affecting its physicochemical and structural properties, contributed an important antioxidant effect for the meat.

## Graphical abstract

Incorporating turmeric into alginate‐based films produced interactions influenced the mechanical and barrier properties and have advantages told over traditional plastic containers.The addition of turmeric increased the antioxidant capacity, and the results indicate great potential to be applied as a packaging material.Edible film added with turmeric reduces the oxidation of lipids and extends the shelf life of pork (12 days), beef (16 days), and chicken (12 days) in refrigeration storage.

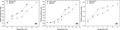

## 1 INTRODUCTION

Meat is one of the foods with the highest protein content and provides essential amino acids needed in the human diet (Baltic & Boskovic, 2015). However, it is susceptible to oxidative deterioration, a limiting factor in quality, since various products such as aldehydes, ketones, alcohols, and other toxic compounds are produced during lipid oxidation (Chang et al., 2018). These substances negatively affect attributes such as color, texture, and flavor (Ribeiro et al., 2019). Theses consequences reduce the shelf life and diminish the nutritional value of the meat (Liu, Xu, Dai, & Ni, 2015), problems that represent a risk for the consumers and economic losses for the producers.

Due to an increased demand for safe products by consumers and the need to reduce the loss of fresh food (Fabra, Falcó, Randazzo, Sánchez, & López‐Rubio, 2018), currently, there is a tendency in the search for biodegradable packaging technologies that contribute to reducing the negative impact on the environment caused by conventional packaging materials (Mkandawire & Aryee, 2018). In this sense, the development of edible films is particularly interesting. They can preserve food and improve their quality and safety by controlling barrier properties (Acevedo‐Fani, Salvia‐Trujillo, Rojas‐Graü, & Martín‐Belloso, 2015).

Edible films on based polysaccharides exhibit excellent barrier properties against oxygen, aromas, and lipids, in addition to adequate mechanical properties (Salgado, Ortiz, Musso, Di Giorgio, & Mauri, 2015). Recent research focused on the development of active and biodegradable packaging through the incorporation of antioxidants and antimicrobials in its formulation, with the purpose of producing edible films with functional properties (Giménez, López de Lacey, Pérez‐Santín, López‐Caballero, & Montero, 2013).

Curcuma (*Curcuma longa* L.) is a rhizome that belongs to the family Zingiberaceae and is widely used as a spice and dye (Martins, Roriz, Morales, Barros, & Ferreira., 2016). It also possesses a broad spectrum of properties, including anti‐inflammatory, antibacterial, antioxidant, nematicide, etcetera (Gupta et al., 2011). Its main component is curcumin, a polyphenol responsible for its antioxidant capacity (Tylewicz, Nowacka, Martín‐García, Wiktor, & Gómez Caravaca, 2018). Due to this antioxidant and antibacterial properties, the use of turmeric extract has been proposed in the aquaculture sector to prevent diseases in fish and thus avoid the use of antibiotics that accumulate in fish meat (Watts, Schreier, Lanska, & Hale, 2017), or as a supplement in feed for broilers chicken to maintain the oxidative stability and physical properties of meat (Kumar et al., 2020). Some studies have proven that the turmeric extract in vitro has a greater antimicrobial and antioxidant effect on fish, compared to herbal extracts such as those from seaweed, *Spirulina*, beets (Devi, Dhayanithi, Kumar, Balasundaram, & Harikrishnan, 2016), and in extracts of ginger and garlic (Tattari et al., 2013). Further, some investigations suggest that turmeric extracts can be applied as natural preservatives in ready‐to‐eat foods (Gul & Bakht, 2015). The use of the turmeric extract has also been explored as nanopolisomes and nanoemulsions loaded, which function as an antioxidant and antimicrobial in fortified beverages and milk as colloidal food models (Karimi et al., 2019; Park et al., 2019). Likewise, it has also been combined with hydrocolloid gums, for improving the oil absorption during deep‐fat frying (Mousa, 2018).

Nowadays, some research focused on the development and characterization of edible films that included curcumin as a bioactive compound, due to its antioxidant and antimicrobial properties (Liu, Cai, Jiang, Wu, & Le, 2016; Ma, Du, & Wang, 2017; Ma, Ren, & Wang, 2017; Roșu et al., 2017). However, until now, no edible films have been developed that included complete turmeric addition, nor has its use been documented for application in fresh meat. The objective of this research was to characterize and evaluate the antioxidant capacity of edible sodium alginate films with turmeric on fresh pork, beef, and poultry to prolong the shelf life of the meat during refrigeration storage.

## 2 MATERIALS AND METHODS

### 2.1 Materials

Reagents were purchased from the following vendors: sodium alginate and propyl gallate (Sigma, St. Louis MO, USA), glycerol (Monarca Additives, Mexico), calcium chloride (PRM, Mexico), food grade turmeric (*C. longa* L.; Alimentaria Mexicana Bekarem, Mexico) and ethylenediaminetetraacetic acid (EDTA), thiobarbituric acid, and hydrochloric acid (J.T. Baker, New Jersey, USA). All the other reagents used in this study were of analytical grade.

### 2.2 Preparation of the edible film with turmeric (EFT)

The EFT‐forming solution was prepared by dispersing alginate and turmeric in distilled water, in a 1.0:0.13 ratio (%, w/w) at 70°C for 30 min under constant stirring. One percent glycerol was added and vigorously mixed for another 30 min. The mixture was poured into polyethylene plates (64 cm^2^) and placed in a dehydrator (Excalibur 4900, Spain) at 41ºC for 12 hr. Finally, 15 ml 1% (w/v) calcium chloride (dry film formulation) was added. Then, they were stored at 53% relative humidity for analyzing physical and mechanical properties.

### 2.3 Physical and structural characterization of the EFT

#### 2.3.1 Thickness

The EFT thickness was measured with a micrometer (Model MDC‐1 "SB," AMES, USA) to the nearest 0.001 mm at five random positions.

#### 2.3.2 Moisture content (MC) and solubility in water (WS)

MC was determined by the EFT weight loss after oven drying at 105°C for 24 hr (ASTM, 2007). WS was determined as described by Gontard, Guilbert, and Cuq (1993), with slight modifications. The MC and WS determinations were both performed in triplicate.

#### 2.3.3 Antioxidant capacity

The EFT antioxidant activity was performed with the 2,2‐diphenyl‐1‐picrylhydracil (DPPH) method, according to Brand‐Williams, Cuvelier, and Berset (1995) and Dashipour et al. (2015). A homogenized sample of 25 mg EFT with 5 ml distilled water was prepared with constant agitation. The extract (0.1 ml) was mixed with 3.9 ml DPPH solution (0.1 mM methanol solution) and incubated for 60 min in the dark at room temperature. The absorbance was measured at 517 nm. The percentage of DPPH radical uptake activity was calculated using equation (1).

(1) $$DPPH\left(\%\right)=\frac{A_{\mathrm{control}}-A_{\mathrm{sample}}}{A_{\mathrm{control}}}x100,$$

where $A_{\mathrm{control}}$ is absorbance obtained from the blank, and $A_{\mathrm{sample}}$ is the absorbance obtained from the sample.

#### 2.3.4 Color

The color was determined with a CR‐400 Minolta Chroma Meter colorimeter (Konika Minolta, Valencia, Spain). The CIELab color scale was used for measurements: L* = 0 (black) to L* = 100 (white), −a* (green), +a* (redness), −b* (blue), and +b* (yellow). The EFT color was measured by placing the sample on the surface of the standard plate. The meat samples were measured immediately after taking them out of their packages.

#### 2.3.5 Transparency

The relative transparency property was measured with a UV‐Vis spectrophotometer (Thermo Fisher Scientific Inc., UK); the readings were performed at 600 nm. The EFT sample was cut into rectangular pieces (4 1 cm) and placed directly in a cell test of the spectrophotometer. The measurements were made using air as a reference. It was calculated with equation (2) (Han & Floros, 1997).

(2) $$\text{Transparency}=A_{600}/\delta ,$$

where A_600_ is the absorbance at 600 nm, and δ is the thickness of the film (mm).

#### 2.3.6 Water vapor permeability (WVP)

WVP was measured gravimetrically using the ASTM E96/E96M‐10 (ASTM, 2010b). Distilled water was placed inside 3.5‐cm‐diameter Payne permeability cups (Elcometer SPRL, Hermelle‐sous‐Argenteau, Belgium); then, film samples were secured with the inward‐facing side exposed to 100% HR. The cups were placed in an equilibrated relative humidity (RH) cabinet at 54% RH at 20°C. They were weighed periodically (± 0.0001 g) until a steady state was reached. WVP was determined as described by Fabra et al. (2017).

#### 2.3.7 Oxygen permeability (OP)

Oxygen permeability obtained from the oxygen transmission rate (OTR) was calculated using an OXTRAN Model 2/21 ml Mocon system (Lippke, Neuwied, Germany). The EFT samples were previously purged with nitrogen at moisture equilibrium before exposure to 10 ml/min oxygen flow. The exposure zone during the test was 5 cm^2^ for each EFT sample. To obtain the OP, the EFT thickness and the difference in the partial pressure of oxygen were considered. The experiments at 23°C and 53% RH were developed.

#### 2.3.8 Mechanical properties

EFT mechanical properties were analyzed in a Mecmesin MultiTest universal test machine (Landes Poli Ibérica, SL, Barcelona, Spain) equipped with a 100 N static load cell. It was used to determine tensile strength (TS), elastic modulus (EM), and elongation at break (EAB), according to the ASTM D882‐091 8 (ASTM, 2010a). TS, EM, and EAB were determined from stress–strain curves, which were estimated from force–distance data obtained for the different samples (1 8 cm). The experiments were performed at 50 mm/min until breaking.

#### 2.3.9 Infrared spectroscopy

EFT structural characterization was examined with a Fourier‐transform infrared spectrophotometer (FTIR; Bruker, Vertex, Wisconsin, USA) using the attenuated total reflectance (ATR) sampling method. Sixty‐four scans were used with a resolution of 4 cm^−1^ in the spectral region of 4000–400 cm^−1^. The spectra were obtained in triplicate.

### 2.4 Preparation of meat samples

The raw meat was purchased from a local market. The pork and beef samples were obtained from the loin muscle (*Longissimus dorsi*) free of fat content; while the chicken samples were acquired from the breast (*Pectoralis major*) and obtained 24 and 8 hr *postmortem*, respectively. The meat was cut in pieces of 10 g (4 × 2 × 1 cm) and fully wrapped with the EFT, and the meat samples without film packaging were considered as the control. A total of 81 samples were prepared for each type of meat and treatment (with EFT and without EFT). The analyses were performed in triplicate. Finally, all samples were stored at 4°C ± 0.5°C for analysis in two‐day intervals, until an acceptable estimated shelf life of meat was reached. Before pH, color, and thiobarbituric acid reactive substances (TBARS) measurements, the film was removed off from meat.

### 2.5 pH

pH of the meat samples was measured with a potentiometer (Thermo Fisher Scientific Inc., UK); a 10 g sample was homogenized with 100 ml distilled water. The extract was filtered using cotton gauze to remove the connective tissue.

### 2.6 Lipid oxidation

The TBARS assay was used to determine lipid oxidation in the meat samples (Tarladgis, Watts, Younathan, & Dugan, 1960). Ten g meat with or without EFT was homogenized with 15 ml double‐distilled water at 50°C and 5 ml of PG‐EDTA (0.5% PG and 0.5% EDTA). Next, 77.5 ml double‐distilled water and 2.5 ml 4 N HCl were added to the extract. The solution was transferred to a Kjeldahl flask and heated with steam distillation until 50 ml condensate was obtained. Five ml of this condensate was removed and mixed with 5 ml 0.02 M 2‐TBA solution to be incubated in boiling water for 35 min. The solution was cooled at room temperature, and the absorbance was measured at 532 nm. The blank contained 5 ml double‐distilled water with 5 ml the 2‐TBA solution. TBARS values were expressed as mg malonaldehyde/kg meat (mg MDA kg^−1^ meat).

### 2.7 Statistical analysis

A completely randomized design was used, with a factorial arrangement in the treatments, the meat factor with three levels (pork, beef, and chicken) and the film factor with two levels (with and without EFT). The data were analyzed under a mixed‐effects model and subjected to a one‐way analysis of variance (ANOVA) using the statistical software SAS® (Statistical Analysis System, USA). The mean tests were performed using Tukey's test. The significance level was set at *p* < .05.

## 3 RESULTS AND DISCUSSION

### 3.1 Characterization of the EFT

**FIGURE 1:**
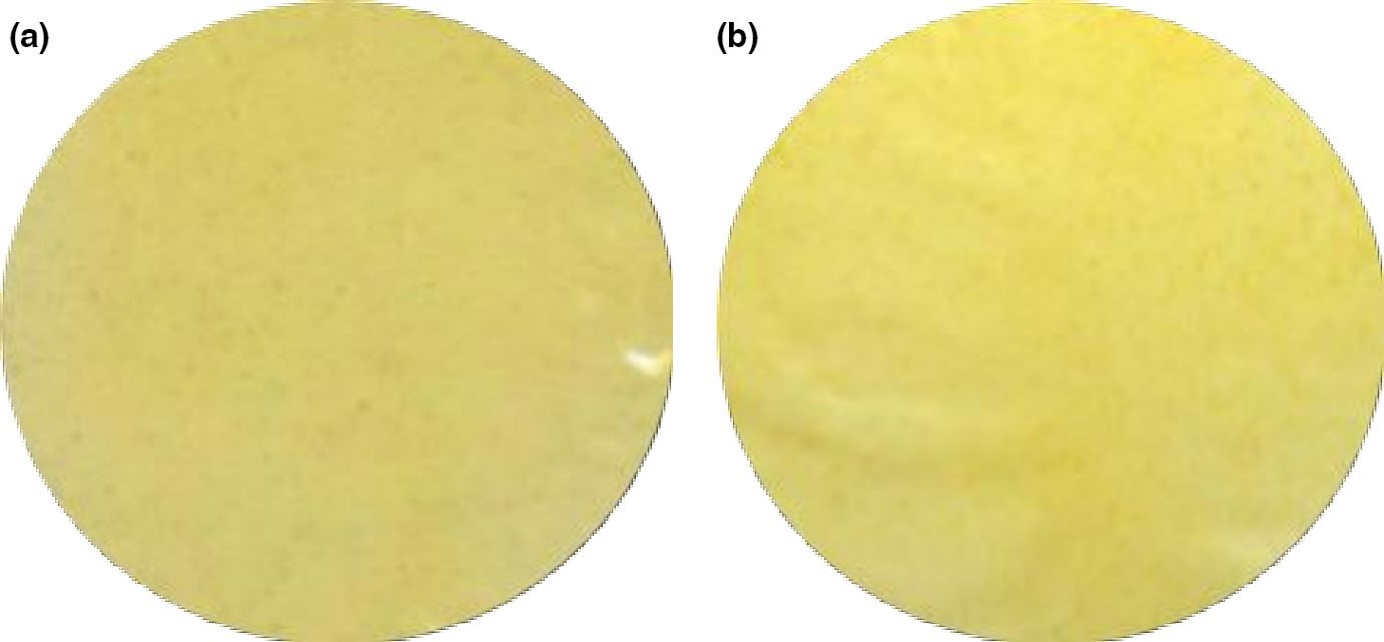
The appearance of the edible film based on alginate with turmeric, in a 1.0:0.13 ratio (%,w/w); (a) front and (b) back

Previous results in the development of the EFT (data not shown) indicated that the higher the amount of added turmeric, the greater the antioxidant activity. However, a 1.0:0.15 ratio (%, w/w) of turmeric did not show statistically significant differences (*p* < .05) in the antioxidant activity compared to a 1.0:0.13 ratio. Addition of higher turmeric concentrations to the edible film was not favorable for its application in meat, a factor closely related to consumers’ purchasing habits and decisions. The optical properties were modified considerably, with a decrease in transparency, an increase in the variables a* and b*, and altered light scattering. Thus, the 1.0:0.13 (%, w/w) alginate/turmeric ratio was chosen for its appearance in the EFT development; it provided a slight yellow color (Figure 1). Additionally, the film exhibited homogeneity and flexibility, aspects that also contributed to its application in meat.

#### 3.1.1 Thickness

The developed EFT thickness was 0.096 mm (Table 1), a value greater than that reported by Musso, Salagado, and Mauri (2017), which was 0.051 mm for a gelatin‐based film with curcumin. This film thickness difference is probably related to the constituents as well as the hydrophobic molecule content of the film‐forming polymer.

**TABLE 1:** Physical and chemical characterization of the edible film based on alginate added with turmeric (1.0:0.13 ratio (%, w/w))

Variables	Edible film
Thickness (mm)	0.096 ± 0.002
Moisture content, MC (%)	23.83 ± 1.05
Solubility, WS (%)	100 ± 0.01
Water vapor permeability, WVP (g mm/kPah^−1^m^−2^)	1.73 ± 0.049
Oxygen permeability, OP (cm^3^mm m^−2^atm^−1^day^−1^)	1.89 ± 0.23
Mechanical properties	
Tensile strength, TS (MPa)	8.26 ± 1.79
Elongation at break, EAB (%)	35.94 ± 2.75
Young's Module, EM (MPa	42.33 ± 1.79
DPPH (%)	38.282 ± 0.85

The film exhibited low MC (23.83%; Table 1), similar to that reported by Musso et al. (2017), with 22.1% in gelatin films with curcumin. On the other hand, Senturk Parreidt, Müller, and Schmid (2018) consider alginate films as sacrificial moisture agents, because the moisture in the food is preserved and the moisture contained in the film evaporates. Nevertheless, Gontard et al. (1993) indicate that amino groups and proteins form hydrogen bonds with hydroxyl (OH) groups of water molecules, which causes greater susceptibility to hydration that influences the solubility, MC, and water permeability of the film. This factor represents a drawback to the film, since effective moisture transfer control is desirable, especially when in contact with fresh meat.

The EFT was completely soluble in water (Table 1). Murad. Karim, Bhat, Uthumporn, and Chew (2011) reported similar results in sodium alginate films; they dissolve in 100% water after 1 min of contact. The high alginate solubility is likely due to its hydrophilic nature, which is determined by alternating units of α‐D‐mannuronic acid and β‐L‐guluronic acid. Fabra et al. (2018) indicated that pure alginate films have certain limitations due to their high solubility under medium–high RH conditions. Although turmeric has very low aqueous solubility, its solubility improves significantly when it binds alginate, as reported by Dey and Sreenivasan (2014), who formed pharmaceutical conjugates of alginate‐curcumin. Thus, since highly soluble alginate‐based films with glycerol and turmeric were incorporated, the solubility of the latter compound improved and resulted in complete film solubility.

#### 3.1.2 Antioxidant capacity

DPPH radicals are widely used to test the antioxidant activity of certain active compounds, such as electron‐donating agents. The function of the antioxidant agent is to form a stable radical and eliminate it by electron donation (Dawidowicz, Wianowska, & Olszowy, 2012). Table 1 shows the EFT antioxidant capacity, estimated from the percentage of DPPH elimination. In general, the EFT antioxidant activity was similar to that obtained by Ma, Ren, et al. (2017), who reported an activity of 35.16% for films made with tara gum/active polyvinyl alcohol and curcumin, and by Kim, Baek, and Song (2018), with 32.96% in alginate films with black chokeberry extract. Yang, Lee, Won, and Song (2016) note that the antioxidant activity of plant extracts is associated with the number of phenolic compounds; that is, a higher extract concentration generally increases DPPH radical uptake. Additionally, phenolic compounds interact with the major component of the matrix, which plays an important role in the functional properties of these films. Further, the present interactions contribute to improve the WVP and, in some cases, reduce solubility (Fabra et al., 2018).

The antioxidant activity of turmeric is mediated by curcumin, mainly by the phenolic hydroxyl; a small fraction may come from the CH_2_ site (Priyadarsini et al., 2003). Under alkaline conditions, the phenolic OH group can be converted to the phenolic oxygen anion, which is a likely reaction site of free radicals that generates a phenyl radical. However, since the film has desirable antioxidant properties, some curcumin phenolic groups were possibly not used to form radicals. Therefore, the EFT is a promising option to prolong the shelf life of food, especially meat. It represents an active container, and the release or absorption of the active component in the food or in the environment is not desirable. Additionally, it can be controlled because it is found in low quantities and not present directly in the meat. It would also help improve food quality, since it would provide protection against color deterioration and lipid oxidation (López‐de‐Dicastillo, Gómez‐Estaca, Catalá, Gavara, & Hernández‐Muñoz, 2012).

#### 3.1.3 Color and transparency

The addition of turmeric dispersed in the film‐forming solution and gave it a yellow coloration. The color was from curcumin, a hydrophobic polyphenol derived from turmeric, which generates a yellow‐orange hue (Almeida et al., 2018). The brightness value was 93.41 ± 0.74, similar to that obtained by Musso et al. (2017), which was 93.3 in gelatin‐based films with 0.02 (%, w/v) curcumin. The a* value for the EFT was 6.53 ± 0.18, similar to that reported by Ma, Du, et al. (2017), with 6.05 in tara rubber films that incorporated curcumin (with a slight orange color). The b* value (degree of yellowness) obtained in the EFT was 43.94 ± 1.60, a value very close to that reported by Wang, Xue, and Zhang (2019), with 40.4 for films with nanoparticles composed of sodium caseinate loaded with curcumin and meina. Priyadarsini (2014) explains that the yellow coloration in film depends on curcumin solubility in water (due to its hydrophobic nature). Active films contain natural pigments that are expressed as colored compounds and induce changes in color parameters (Bitencourt, Fávaro‐Trindade, Sobral, & Carvalho, 2014). This phenomenon indicates that the addition of 0.13% turmeric to the edible film significantly impacted light scattering, a fact that was confirmed in the appearance, transparency, and color that the film adopted.

On the other hand, the EFT transparency was 7.78%, a value higher than that obtained by Ramos et al. (2012). In that study, the values oscillated between 1.35% and 3.09% in films based on whey protein isolate. This discrepancy is probably due to the fact that curcumin, a diaroylmethane responsible for the characteristic yellow color in turmeric, exhibits lower opacity of light transmission (Gürses, 2019). Fabra et al. (2018) note extracts that contain polyphenols tend to selectively absorb light, especially at low wavelengths, a phenomenon that imparts color to the edible film and increases opacity. This change provides a protective barrier against UV light that could contribute to decreased meat deterioration and prevent the oxidation of myoglobin and the lipids present in the food.

#### 3.1.4 Water vapor permeability

The WVP in an edible film is a parameter that demonstrates the transfer of moisture in the material and in the food. WVP is dependent on the type of polymer, structural composition of alginate (mannuronic acid/guluronic acid), concentration of the cross‐linked agent, and storage conditions such as RH and temperature. Therefore, in food packaging, it is desirable that the WVP value be as low as possible to avoid moisture loss in food (Gontard, Guilbert, & Cuq, 1992). The WVP value for the EFT was low (1.73 g mm/kPah^−1^m^−2^; Table 1) compared with that reported by Gholizadeh, Buazar, Hosseini, and Mousavi (2018) in alginate films that contained hydroxyapatite nanoparticles (11.85– 23.17 g mm/kPah^−1^m^−2^). Rangel‐Marrón, Mani‐López, Palou, and López‐Malo (2019) showed that in alginate films with mashed papaya added with citric acid, the WVP value was 0.002 g mm/kPah^−1^m^−2^. These values could be related to the polarity, solubility, and diffusivity of the water molecules present in the composition of hydrophilic films, a fact that significantly impacts WVP (Zhang, Xu, Gao, & Fu, 2013). Further, a decrease in WVP occurs due to cross‐linking, which modifies the film permeability (Costa et al., 2018).

Curcumin has a long chain of carbon and hydrophobicity in the benzene rings that decreases the WVP value (Wang et al., 2019). Nevertheless, Musso et al. (2017) found that the hydrophobic nature of curcumin causes a poor dispersion that agglomerates it and alters the compact structure of the film, factors that decrease WVP. This fact could be associated with hydrophobic interactions and the formation of hydrogen bonds, which restrict the number of free OH groups to interact with the water present in the edible film (Rashidinejad, Birch, Hindmarsh, & Everett, 2017). This behavior hinders water movement in the film, a phenomenon that indicates water vapor transfer obeys the percentage of hydrophilic composition in the film (Gomaa, Fawzy, Hifney, & Abdel‐Gaward, 2018).

A high dispersion in the WVP values reported in edible films indicates a relationship between the chain structure of the polymer and the distribution of its components in the matrix. In other words, a poorly ordered structure could allow greater interactions between the polymers and water molecules (Letendre, D'Aprano, Delmas‐Patterson, & Lacroix, 2002). Likewise, in polysaccharides such as alginate, WVP tends to increase when there is a high humidity difference between the environment and the food (Hambleton, Debeaufort, Beney, Karbowiak, & Voilley, 2008). This fact could be inconvenient for foods with high moisture content (e.g., meat) that are exposed to high RH when stored under refrigeration. This phenomenon is because the water behaves like a plasticizer in hydrophilic films and causes the movement of diffusing molecules, actions that lead to film swelling and an increase in WVP (Hambleton, Perpiñan‐Saiz, Fabra, Voilley, & Debeaufort, 2012).

#### 3.1.5 Oxygen permeability

OP in edible films has a direct effect on the quality and shelf life of the food, so a lower OP value represents better barrier capacity against oxygen in the packaging material (Ma, Ren, et al., 2017). The OP value obtained was relatively low (Table 1) compared with those obtained by Ma, Du, et al. (2017), which ranged between 7 and 8.51 cm^3^ mm m^−2^atm^−1^día^−1^ day^‐1^ for films that contained curcumin, and from 42.7 to 187 cm^3^ mm m^−2^ atm^−1^día^−1^ day^‐1^ for commercial high‐density and low‐density polyethylene films.

These results may be associated with bond interactions between curcumin and the carbohydrate matrix, which can prolong or limit oxygen access (Liu et al., 2018). Generally, phenolic compounds that are incorporated into carbohydrate‐based films have the capacity to form hydrogen bonds between the carboxyl groups, a phenomenon that induces a cross‐linking effect that decreases OP (Fabra, Hambleton, Talens, Debeaufort, & Chiralt, 2011). Overall, the EFT presented a good barrier against oxygen, even better than commercial plastic films.

#### 3.1.6 Mechanical properties

The values for TS, EAB, and Young's modulus are shown in Table 1. The TS value for the EFT was 8.26 MPa, slightly higher than that obtained by Wang & Zhang (2019), which was 7.4 MPa in NaCas/zein films loaded with curcumin. These data indicate that the EFT was stronger in order to support extensibility by external tensile stress before breaking. Kalaycıoğlu, Torlak, Akın‐Evingür, Özen, and Erim (2017) observed that the addition of turmeric extracts in chitosan films increases the TS value 1.5 times compared to film without the extract. This resistance depends on the polymer, the internal union, and the possible interactions between the matrix and the active compound (Gilbert, Cheng, & Jones, 2018).

For EAB, the EFT elongation was 35.94%, higher than that reported by Ma, Ren, et al. (2017) and Wang & Zhang (2019), which are 29.88 and 30.8% in tara gum films added with curcumin and caseinate/zein films with curcumin, respectively. Bitencourt et al. (2014) observed that at 50 g of turmeric ethanol extract/100g of gelatin (a higher concentration than used in this study), the EAB value significantly increases. Noronha, de Carvahlo, Lino, and Barreto (2014) indicate that the presence of phenolic compounds in the film could increase EAB, potentially due to interactions between curcumin and alginate, a phenomenon that would generate a more flexible and cohesive matrix.

For Young's modulus value, although there are no determined values for each food, a high value indicates that a material is strong. In this study, the EFT value was 42.33 ± 1.79 MPa, which was lower compared to that reported by Kalaycıoğlu et al. (2017) in chitosan films with turmeric extract (2064 ± 155 MPa) and with turmeric flour films from the dye extraction residue (values obtained between 246.8 ± 46.8 and 875 ± 15.7 MPa; Maniglia, de Paula, Domingos, & Tapia‐Blácido, 2015). This result could be due to the hydrophobicity of the matrix caused by the incorporation of turmeric, which causes changes in the cross‐linking and decreases Young's modulus.

Overall, the goal is to develop flexible films that adhere perfectly to the surface of the food, as a "second layer," to preserve the shape, texture, and integrity of the product (Rosa, 2019). The EFT has the potential to be applied to meats as a packaging system that maintains properties such as appearance, color, water retention capacity, lipid stability, and microbial spoilage.

#### 3.1.7 Infrared spectroscopy

**FIGURE 2:**
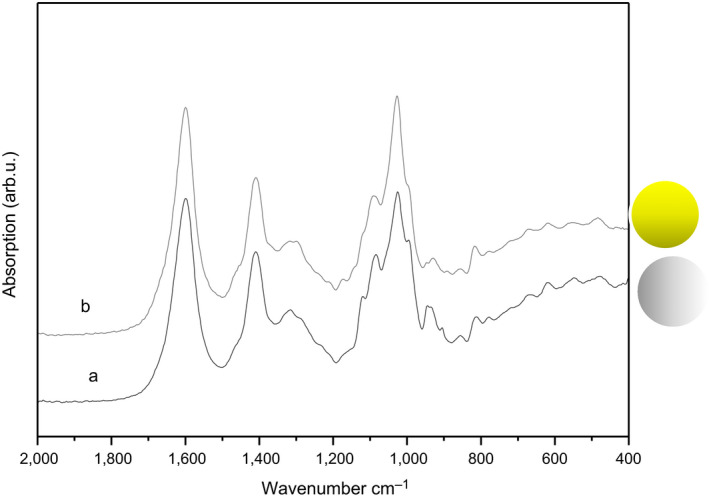
Infrared spectra of the alginate films (a) without turmeric and (b) with turmeric in 1.0:0.13 ratio (%, w/w)

The infrared spectroscopy technique was used to analyze molecular changes mediated by the addition of turmeric. Figure 2 shows the spectra of the alginate films with and without turmeric. The line shapes were very similar, and there were only slight band shifts. Therefore, the lack of observable drastic changes suggests that the chemical stability of the film is not compromised by the presence of turmeric. In general, both spectra showed bands associated with the functional groups C = O, C‐O‐C, and COOH (all present in the alginate spectrum). Additionally, the bands associated with the presence of the turmeric had low absorption values (Figure 2b), because turmeric is present at a lower concentration.

### 3.2 EFT application effects on meat

#### 3.2.1 Determination of pH value

Meat pH is related to quality and freshness characteristics, such as color, tenderness (Jayasena et al., 2013), microbial growth, and the addition of antioxidants (Battisti et al., 2017). EFT significantly affected the pH of pork and beef (Table 2; *p* < .05). The pH value in the meat, with and without EFT, during refrigeration storage increased gradually, but in those with EFT, this increase was lower. In chicken breast meat, there were no significant differences in pH with or without EFT (Table 2; *p* < .05).

**TABLE 2:** Physical and chemical analysis of fresh pork, beef (*Longissimus dorsi*), and chicken meat (*Pectoralis major*), stored in refrigeration at 4°C with and without edible film added with turmeric (1.0:0.13 ratio (%, w/w))

Variable	EFT					
	Pork		Beef		Chicken	
	Without	With	Without	With	Without	With
Refrigeration Storage (4°C) (d)	8	12	12	16	8	12
pH	6.37 ± 0.10^a^	6.11 ± 0.15^c^	6.45 ± 0.10^a^	6.21 ± 0.10^b^	6.38 ± 0.10^a^	6.45 ± 0.14^a^
Color (Lab*)						
L*	54.39 ± 0.12^a,b^	54.43 ± 0.15^a,b^	53.07 ± 0.15^b^	55.08 ± 0.20^a^	47.45 ± 0.10^c^	35.96 ± 0.12^d^
a*	3.09 ± 0.10^c^	3.06 ± 0.05^c^	9.31 ± 0.11^a^	9.39 ± 0.10^a^	1.82 ± 0.11^d^	6.19 ± 0.10^b^
b*	9.88 ± 0.05^c,d^	8.99 ± 0.05^c^	12.91 ± 0.05^c^	12.86 ± 0.05^c^	20.12 ± 0.10^b^	30.96 ± 0.10^a^
TBARS (mg MDAkg^−1^ meat)	0.30 ± 0.01^a,b^	0.30 ± 0.01^b,c^	0.33 ± 0.04^a,b.c^	0.28 ± 0.01^c^	0.31 ± 0.01^b,c^	0.37 ± 0.01^a^

Note

The values reported are their means (*n* = 3) ± standard deviation. Different letters in the same row indicate significant differences between the samples (*p* < .05), according to the Tukey test.

The increase in pH during storage can be attributed to protein denaturation and the accumulation of alkaline byproducts such as ammonia, amines, and trimethylamine, all of which are produced during amino acid degradation by autolytic or microbial reactions (Lorenzo, Batlle, & Gómez, 2014). Likewise, the pH tendency in meat with EFT is probably due to the phenolic compounds present in the edible film and its inhibitory effect on microbial growth during storage (Ehsani, Jasour, Hashemi, Mehryar, & Khodayari, 2014). Indeed, curcumin has antibacterial functions in addition to its antioxidant effects (Chan, Ng, Tan, & Low, 2011).

The pH results in pork with EFT were similar to minced pork wrapped in gelatin film with catechin–lysozyme (Kaewprachu, Osako, Benjakul, & Rawdken, 2015); the wrapped pork exhibited a pH increase from 5.7 to 6.4 during 4 days of storage. In beef with edible film, Amiri, Aminzare, Azar, and Mehrasbi (2019) reported similar results in fresh ground beef patties wrapped with corn starch films with *Zataria multiflora* essential oil (thyme and oregano) for 20 days of storage. Radha krishnan et al. (2015) reported pH values of 5.80–6.23 after 15 days of storage for samples of beef wrapped in corn starch films with clove and cinnamon essential oils. Pirsa and Shamusi (2019) observed similar behavior in pH for chicken meat, with a value of 7.38 after 10‐day storage with cellulose‐polypyrrole‐ZnO films.

#### 3.2.2 Color

Changes in meat color during storage are important to consumers’ acceptance of and decision to buy the product. The mean values of CIE L*, a*, and b* of the meat with and without EFT during storage are shown in Table 2. Notably, all beef and chicken samples were significantly affected (*p* < .05) in the L* value. This effect may be due to the oxygen barrier property of the EFT, which may have delayed oxygen diffusion and its reaction with myoglobin. In beef, the luminosity value increased, while the opposite effect was observed in chicken in the presence of the EFT. This discrepancy could be explained by factors such as the composition of glycolytic fibers in the muscle that is generally associated with greater luminosity (Kim, Cho, & Han, 2013) and the degree of association of myoglobin with oxygen that is related to the muscle pH. Where there is the highest degree of association, the pH value is lower, and the meat will appear "bright" (Hoving‐Bolink et al., 2000). The pork values were not different with or without the EFT.

Low a* values indicate a loss in the redness of the meat, which is associated with the oxidation of myoglobin to metmyoglobin (Warner, 2014). Specifically, an increase in oxymyoglobin causes negative color changes and is generally related to a reduction in freshness (Serrano‐León et al., 2018). This discoloration is strongly linked to the myoglobin content in the muscles; pork has a type of oxidic‐glycolytic muscle IIX, and beef contains an oxidative muscle type I with a high myoglobin content (Astruc, 2014). However, prolonged storage times cause the iron in the heme ring to oxidize to the reduced ferric state (MbFe^3+^), a phenomenon that induces browning of the meat (Baron & Andersen, 2002). In this study, there were no significant differences with or without EFT in pork and beef during refrigerated storage, data that suggest the effectiveness of using EFT to maintain color integrity in this type of meat. In chicken with EFT, the a* value increased, since the chicken breast, being mostly type B fibers, contains relatively little myoglobin (Nishida & Nishida, 1985).

The mean b* values during storage in pork and beef samples with and without EFT were not significantly different. However, for chicken with EFT (Table 2), b* significantly increased (*p* < .05). This change may be caused by the presence of curcuminoids (yellow color) in the edible film. Zhang and Guo (2016) found that the incorporation of spice extracts increases yellowing in fresh chicken meat.

#### 3.2.3 Lipid oxidation

**FIGURE 3:**
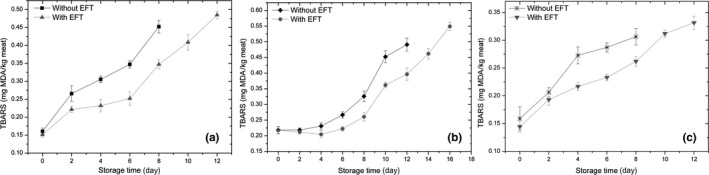
Effect of edible film with turmeric (EFT) on TBARS values in (a) pork, (b) beef, and (c) chicken, under refrigerated conditions at 4°C

The mean TBARS values of the meat with and without EFT are presented in Table 2. Overall, the EFT produced a significant effect in the different meat. The change in TBARS values during refrigeration storage in pork (a), beef (b), and chicken (c) is presented in Figure 3. The TBARS value increased significantly in all cases, results that demonstrate increased lipid oxidation during storage. Lee et al. (2010) indicate that the storage period significantly influences lipid oxidation in meat. However, the TBARS analysis showed that pork, beef, and chicken with EFT had significantly lower TBARS values compared to those without EFT (*p* < .05).

Of all the meat, pork showed the highest TBARS values during refrigeration storage, although it remained below 0.5 mg MDA kg^−1^ meat for 12 and 8 days with and without EFT, respectively. Zhang, Wu, and Guo (2016) indicated that TBARS values between 0.202 and 0.664 mg MDA kg^−1^ meat in pork represent fresh meat. Cheng, Liu, Zhang, Chen, and Wang (2018) showed that at 5 mg MDA kg^−1^ meat, rancidity is already detectable. In this study, beef with EFT had an oxidation level of 0.6 mg MDA kg^−1^ meat after 16‐day storage, a lower TBARS value compared to Navikaite‐Snipaitiene et al. (2018), with 0.8–0.96 mg MDA kg^−1^ meat after 14‐day storage in an antioxidant package with eugenol. Similarly, Kim, Jeong, et al. (2013) obtained TBARS values between 0.98 and 1.11 mg MDA kg^‐1^ meat in patties treated with 0.1% extracts of *Pimpinella brachycarpa* (Kom.) Nakai for 12 days in refrigerated storage. In chicken with EFT, on day 12 of storage, the TBARS value was 0.33 mg MDA kg^−1^ meat, a lower value than that reported by Giteru, Oey, Ali, Johnson, and Fang (2017) for chicken fillets (0.6 mg MDA kg^−1^ meat after 96‐hr refrigerated storage in kafirin films that incorporated citral). Fernández, Pérez‐Álvarez, and Fernández‐López (1997) estimated that TBARS levels ≥ 1 mg MDA kg^−1^ meat are the limit at which consumers detect flavors and strange smells in the meat.

The obtained results show that EFT was effective against lipid oxidation in all the meat tested during refrigeration storage, because it significantly reduced TBARS formation (*p* < .05). The inhibitory effect of oxidation in meat with EFT may be due to its ability to block oxygen and the antioxidant property of turmeric, especially due to its high content of curcuminoids (polyphenols; Pulido‐Moran, Moreno‐Fernandez, Ramirez‐Tortosa, & Ramirez‐Tortosa, 2016). This finding is corroborated by Abdou, Galhoum, and Mohamed (2018), who indicated that the inclusion of curcumin in nanoemulsion coatings reduces TBARS values in chicken, a phenomenon that could inhibit MDA formation. Similarly, Fernandes, Trindade, Lorenzo, Munekata, and de Melo (2016), Krishnan et al. (2014), and Lorenzo et al. (2018) indicate that the inclusion of natural extracts rich in phenolic compounds helps to delay lipid oxidation in meats.

## 4 CONCLUSION

Edible films have attributes beyond being biodegradable and biocompatible, advantages they hold over traditional plastic containers. Incorporating turmeric into alginate‐based films produced interactions between turmeric compounds and the polymer matrix. These interactions influenced the mechanical and barrier properties. The addition of turmeric increased the antioxidant capacity, results that indicate great potential to be applied as a packaging material and extend the shelf life of pork (12 days), beef (16 days), and chicken (12 days) in refrigeration storage, with a particular benefit to reducing lipid oxidation. Consequently, the development of the EFT is proposed as a promising technology to increase shelf life and preserve its quality in different meat.

## CONFLICT OF INTEREST

The authors have declared no conflict of interest for this article.
